# Development of a personal heat strain risk assessment (PHSRA) index in workplaces and its validation

**DOI:** 10.1186/s12889-020-08874-4

**Published:** 2020-06-03

**Authors:** Saeid Yazdanirad, Farideh Golbabaei, Mohammad Reza Monazzam, Habibollah Dehghan, Abbas Rahimi Foroushani

**Affiliations:** 1 Department of Occupational Health Engineering, School of Public Health, Tehran University of Medical Sciences, Tehran, Iran; 2 Department of Occupational Health Engineering, School of Public Health, Isfahan University of Medical Sciences, Isfahan, Iran; 3 Department of Epidemiology and Biostatistics, School of Public Health, Tehran University of Medical Sciences, Tehran, Iran

**Keywords:** Heat stress, Risk assessment, Personal factors, Main factors

## Abstract

**Background:**

There is not a comprehensive heat stress index to screen the people susceptible to heat disorders and illnesses in hot workplaces. The present study was aimed to develop a personal heat strain risk assessment (PHSRA) index in workplaces and validate it.

**Methods:**

This cross-sectional study was carried out on 201 Iranian male employees under various thermal conditions. At first, the demographical data of participants were gathered. After that, the heart rate and tympanic temperature of the subjects were carefully measured at times of 30, 60, and 90 min of starting the work. Environmental factors were measured simultaneously. The metabolism rate and insulation value of clothes were also estimated. At the end, a novel index of the heat strain was developed using structural equation modeling in AMOS and validated using linear regression analysis in SPSS.

**Results:**

Indirect effect coefficients of personal factors including age, body mass index, maximum aerobic capacity, and body surface area were equal to 0.031, 0.145, − 0.064, and 0.106, respectively. The coefficients of main factors including dry temperature, wet temperature, globe temperature, wind speed, metabolism, and clothing thermal insulation were obtained as 0.739, 0.688, 0.765, 0.245, 0.482, and 0.383, respectively. These coefficients and normalized values of the factors were used to develop a novel index. The total score of the index was categorized into four levels by optimal cut-off points of 12.93, 16.48, and 18.87. Based on the results of regression analysis, this index justifies 77% of the tympanic temperature as a dependent variable (R^2^ = 0.77).

**Conclusions:**

In general, the results indicated that the novel index developed by the personal and main factors had proper validity in the prediction of thermal strain.

## Background

Approximately one-third of the world’s population is frequently exposed to inappropriate climatic conditions [1]. Heat threats the human health in workplaces, particularly. The thermal exposure causes severe illnesses in addition to milder disorders such as heat rash, heat cramps, heat exhaustion, and heat syncope. Heat stress can be associated with fatal outcomes if it is sustained [2]. As well as, the studies found that there are significant relationships between heat exposure and psychological, safety, socio-economics, and productivity consequences [3]. Ramsey et al. [4] reported that workplace thermal conditions had a significant effect on the safety-related behavior of workers and increased the risk of accidents. Kjellstrom et al. [5] concluded that heat stress could decrease work performance and potentially generate enormous economic consequences. Tawatsupa et al. [6] concluded that heat stress might indirectly cause psychological distress because of decreased productivity, reduced income, and disrupted social activity. Various factors can be effective in producing excessive thermal strain. Intense physical activity increases heat production, and warm climatic conditions or clothing ensembles decrease heat loss during the work [7]. The main factors consist of four climatic (dry temperature, radiant temperature, humidity rate, and air movement (and two non-climatic)clothing and physical workload(variables [8]. However, there are other factors to determine the personal difference in the occurrence of heat-related disorders and illnesses. Some of them include age, obesity, aerobic capacity, and body surface area. The results of a review study conducted by Kenny et al. [9] showed that the subjects aged 60 and more had higher rates of illness, injury, and death in warm environments. Yazdanirad et al. [10] concluded that the mean heart rate of the subjects with overweight and obesity (BMI > 25 kg/m^2^) was significantly higher than that of the subjects with normal weight (BMI < 25 kg/m^2^). Hansen et al. [11] also found that some genetic and physiological factors could reduce the human body resistance, making the subjects susceptible to heat disorders and illnesses. The results of a study carried out by Merry et al. [12] indicated that aerobic fitness attenuated the physiological effects during the exercise under warm and humid conditions. Coso et al. [13] have shown that body mass and body surface area have a negative relationship with core temperature. Havenith et al. [14] concluded that there were significant positive correlations between body surface area per mass and core temperature in warm-humid, mild, and hot-dry environments. These properties affect the thermal strain in various people. However, known heat stress indices such as wet bulb global temperature (WBGT) and predicted heat strain (PHS) indices do not consider them. Hence, these cannot be applied to screen people susceptible to heat disorders and illnesses. A few studies have attempted to develop a heat stress index using personal factors. Lu et al. [15] made a body characteristic index by variables of maximal oxygen uptake per body mass, body surface area per body mass, and percentage of body fat for assessing the thermal strain. Glass et al. [16] also estimated risk of heat strain by variables of age and gender in a simulation model. These indices have no considered the main factors, such as environmental parameters, in computing the risk. Therefore, the present study was aimed to develop a personal heat strain risk assessment (PHSRA) index in workplaces and validate it.

## Methods

### Participants

This cross-sectional study was carried out on 201 Iranian male employees, including 111 subjects from a steel factory as a hot-dry ambiance and 90 subjects from a petrochemical factory as a hot-humid environment. At first, the various parts of the above industries were carefully visited, and desired duties were selected. After that, the characterization of 400 individuals employed in these duties, including age, weight, height, and history of diseases, was extracted from their medical records. Based on this information and criteria, 199 persons were excluded, and 201 subjects were entered into the study. So that extensive ranges of variables values were involved. Inclusion criteria included the absence of mental, infectious, pulmonary, cardiovascular, renal, and digestive diseases, and lack of diabetes, hyperthyroidism, and hypertension. Other criteria were non-consumption of medications influencing on heart rate and blood pressure, and non-consumption of coffee, caffeine, and alcohol from 12 h before the research. Moreover, their tympanic membrane and auditory canal were examined. Exclusion criteria also consisted of non-cooperation in precise measurement of physiological parameters, feeling of fatigue, heart rate over maximum permissible value computed by eq. 1, and body temperature above 39 °C.

1 $$ HRmax\ \left(\mathrm{beat}/\min \right)=\left[208-\left(0.7\times age\ (year)\right)\right] $$

### Sample size calculation

The present study was aimed to develop a novel index using factors affecting body temperature. The minimum probable correlation between the novel index and body temperature was considered by 0.2, the sample size with a confidence level of 95% and a test power of 80% was determined as follow [17]:

2 $$ n={\frac{\left({Z}_{1-\frac{\alpha }{2}}+{Z}_{1-\beta}\right)}{w^2}}^2+3\cong 194 $$

Where ${\mathrm{Z}}_{1-\frac{\upalpha}{2}}$ is equal to 1.96 based on a confidence level of 95%, Z_1 − β_ is equal to 0.84 based on a test power of 80%, and W is equal to 0.203 based on a minimum correlation coefficient of 0.2. These values were extracted from related tables [17].

Therefore, the sample size in the present study was suggested to be more than 194 subjects.

### Study design and setting

Data were collected between April and September 2019. At first, the participants were asked to sign the consent form. Later, demographical data of them, including age, height, weight, job, physical activity, and work experience, were gathered. After that, the subjects were invited to rest on a bed in a quiet and temperate room for 30 min. Their heart rate and tympanic temperature were appropriately monitored at times of 20, 25, and 30 min based on the standard of ISO 9886 [18]. At the next step, heart rate and tympanic temperature of the subjects were carefully measured at times of 30, 60, and 90 min of starting the work. Environmental factors including dry temperature (T_a_), wet temperature (T_w_), globe temperature (T_g_), and wind speed (V_a_) were simultaneously measured based on standards of ISO 7243 [19] and ISO 7726 [20]. The metabolism rate was estimated by the standard of ISO 8996 [21] and corrected by the standard of ISO 7243 [19]. The insulation value of clothes (I_c_) was also determined using the standard of ISO 9920 [22]. After ending the measuring process, values of the body mass index (BMI) (kg/m^2^), maximum aerobic capacity (VO2max) (ml/(kg·min)), and body surface area (BSA) (cm^2^) were calculated by eqs. 3, 4, and 5, respectively.

3 $$ BMI=\left(\frac{W_b}{H^2}\right) $$

Where W_b_ is body weight (kg), and H is body height (m) [23].

4 $$ VO2\max =15\times \left(\frac{HRmax}{HRrest}\right) $$

Where HRmax is the maximum permissible heart rate computed by eq. 1 (beat/min), and HRrest is heart rate during resting (beat/min) [24].

5 $$ BSA=71.3989\times {H}^{0.7437}\times {W}_b^{0.4040} $$

Where H is body height (cm), and W_b_ is body weight (kg) [25].

### Measurement instruments

The tympanic temperature was measured using the thermometer of Braun IRT 6530 with an accuracy of 0.1 degrees centigrade. This device, with pre-warmed tips and an exact positioning system, has been registered as a patent. Navarro et al. [26] resulted that Braun thermoscan IRT 6530 accurately estimated the body temperature of subjects during exercise under warm conditions. The heart rate was measured using a pulse monitor of Beurer PM70 with an accuracy of one beat per minute. Factors of dry temperature, wet temperature, and globe temperature were also measured using WBGT meter of TES 1369B with an accuracy of 0.1 degrees centigrade. Wind speed was separately measured using TES 1340 with an accuracy of 0.01 m per second. To measure body height and weight, a tape meter with an accuracy of 0.01 m and a digital scale of Hamilton with an accuracy of 0.1 kg were applied, respectively.

### Index development

After collecting data, the indirect effect coefficients of the factors were computed using structural equation modeling (SEM) [27]. As well as the value of each variable was normalized between 0 and 1 follow as:

6 $$ v\left(i,n\right)=\frac{\left[v(i)-v\left(i,0\right)\right]}{v\left(i, ref\right)} $$

where *v*(*i*, *n*) is normalized variable, *v*(*i*, 0) is the minimum measured value of the variable, and *v*(*i*, *ref*) is the maximum measured value minus the minimum measured value of the variable.

Equation 6 was exploited to calculate the novel index.

7 $$ PHSRA=\left[\left({C}_1\times {A}_n\right)+\left({C}_2\times {BMI}_n\right)+\left({C}_3\times {VO}_{2n}\mathit{\max}\right)+\left({C}_4\times {BSA}_n\right)+\left({C}_5\times {T}_{an}\right)+\left({C}_6\times {T}_{wn}\right)+\left({C}_7\times {T}_{gn}\right)+\left({C}_8\times {V}_{an}\right)+\left({C}_9\times {M}_{tn}\right)+\left({C}_{10}\times {I}_{cn}\right)\right] $$

Where *C*_1_ to *C*_10_ are indirect effect coefficients, A_n_ is normalized age, BMI_n_ is normalized body mass index, VO_2n_max is normalized maximum aerobic capacity, BSA_n_ is normalized body surface area, T_an_ is normalized dry temperature, T_wn_ is normalized wet temperature, T_gn_ is normalized globe temperature, V_an_ is normalized wind speed, M_tn_ is normalized total metabolism, and I_cn_ is normalized insulation of clothes.

### Statistical analyses

Data were entered into the statistical package for the social sciences (SPSS) version 18 [28]. Skew and kurtosis curves were used to examine the normality of variables. The results showed that the statistical distribution of all factors was normal. Therefore, correlations were computed using the Pearson test. After that, a theoretical model was depicted in AMOS software [27]. The fitness of the proposed model was examined using absolute, comparative, and normed fit indices. The indirect effect coefficients of the factors were extracted from the model and used to develop the novel index. Finally, the index score was classified into four levels using Receiver operator curves (ROC) analysis. Body temperatures of 37.5, 38.0, and 38.5 ^0^ C were considered as boundaries of risk levels [29]. Nearest points to ideal state in ROC curves were adopted as optimal cut-off points of the novel index. The validity of the index was also examined by linear regression analysis.

## Results

This study results from a field survey involving 201 subjects, all male, where 111 were employees on a steel factory (hot-dry ambiance) and 90 on a petrochemical factory (hot-humid environment). Table 1 describes the statistical distribution of studied variables, including personal factors, main factors, and physiological parameters. The results showed that the extensive ranges of values were collected. The correlation matrix of the variables has been presented in Table 2. Of personal factors, maximum aerobic capacity had the highest correlation with tympanic temperature. Of the main factors, the most significant correlations were related to environmental variables.

**Table 1:** The statistical distribution of studied variables

Variable		Range	Mean	Standard deviation
Personal factors	Age (year)	22.00–55.00	36.62	8.24
	Body mass index (kg/m^2^)	19.23–34.94	26.06	4.07
	Maximum aerobic capacity (ml/(kg·min))	29.30–44.16	36.16	3.20
	Body surface area (m^2^)	1.64–2.47	1.96	0.17
Main factors	Dry temperature (°C)	21.97–48.20	24.78	6.01
	Wet temperature (°C)	12.10–37.57	22.01	6.60
	Globe temperature (°C)	23.40–62.43	39.89	9.73
	Wind speed (m/s)	0.00–4.20	0.58	0.39
	Total metabolism (watts)	130.00–490.00	248.47	103.70
	Clothing thermal resistance (clo)	0.50–1.35	0.83	0.14
Physiological parameters	Resting tympanic temperature (°C)	36.70–37.40	37.01	0.15
	Working tympanic temperature (°C)	36.70–39.10	37.70	0.56
	Resting heart rate (beat/min)	64.00–94.00	76.14	5.99
	Working heart rate (beat/min)	70.00–189.00	1.21	28.45

**Table 2:** Correlation matrix of the variables

Variable	1	2	3	4	5	6	7	8	9	10	11
Age	–										
Body mass index	0.242^**^	–									
Maximum aerobic capacity	−0.515^**^	−0.497^**^	–								
Body surface area	0.060	0.817^**^	−0.327^**^	–							
Dry temperature	−0.019	0.093	−0.211^**^	0.049	–						
Wet temperature	0.010	−.027	−0.172^*^	−0.065	0.626^**^	–					
Globe temperature	0.009	0.082	−0.151^*^	0.056	0.907^**^	0.511^**^	–				
Wind speed	0.014	0.051	−0.107	0.068	0.296^**^	0.152^*^	0.347^**^	–			
Total metabolism	−0.026	0.060	−0.085	0.004	0.318^**^	0.131	0.332^**^	0.082	–		
Clothing thermal insulation	−0.102	0.017	−0.036	0.048	0.454^**^	0.054	0.530^**^	0.135	0.321^**^	–	
Tympanic temperature	0.139^*^	0.177^*^	0.350^**^	0.101	0.751^**^	0.685^**^	0.766^**^	0.258^**^	0.509^**^	0.388^**^	–

(1) Age, (2) body mass index, (3) maximum aerobic capacity, (4) body surface area, (5) dry temperature, (6) wet temperature, (7) globe temperature, (8) wind speed, (9) total metabolism, (10) clothing thermal insulation, (11) Tympanic temperature.

^**^P <  0.01

^*^P <  0.05

**Fig. 1:**
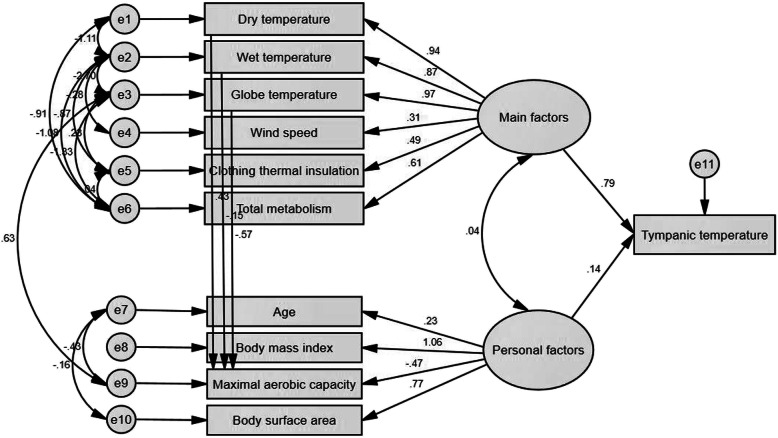
The theoretical model analyzed by SEM

Figure 1 illustrates the theoretical model analyzed by SEM. The results revealed that the main factors with a total coefficient of 0.79 and personal factors with 0.14 had a significant direct effect on the tympanic temperature. Table 3 reports the effect coefficients of the variables in producing thermal strain. Of personal factors, the body mass index and body surface area with coefficients of 0.145 and 0.106 had the highest indirect effects on the tympanic temperature, respectively. Of the main factors, the highest indirect effects belonged to environmental variables, including globe temperature with a coefficient of 0.765, dry temperature with 0.739, and wet temperature with 0.688. Table 4 presents the goodness-of-fit indices of the analyzed model. Based on the results, the fitness of the model was confirmed.

**Table 3:** Effect coefficients of the variables in producing thermal strain

Variable		Direct effect	Indirect effect	*P* value
Personal factors	Age	0.226	0.031	*P* < 0.001
	Body mass index	1.057	0.145	*P* < 0.001
	Maximum aerobic capacity	−0.466	− 0.064	*P* < 0.001
	Body surface area	0.775	0.106	*P* < 0.001
	Thermal strain	0.137	–	*P* < 0.001
Main factors	Dry temperature	0.936	0.739	*P* < 0.001
	Wet temperature	0.872	0.688	*P* < 0.001
	Globe temperature	0.969	0.765	*P* < 0.001
	Wind speed	0.311	0.245	*P* < 0.001
	Total metabolism	0.611	0.482	*P* < 0.001
	Clothing thermal insulation	0.485	0.383	*P* < 0.001
	Thermal strain	0.789	–	*P* < 0.001

**Table 4:** Goodness-of-fit indices of the analyzed model

Index	Name	Threshold of Fitness	Obtained value
Absolute fitness indices	Goodness-of-fit index (GFI)	> 0.9	0.967
	Adjusted goodness-of-fit index (AGFI)	> 0.9	0.920
Comparative fitness indices	Normed fit index (NFI)	> 0.9	0.971
	Comparative fit index (CFI)	> 0.9	0.991
	Incremental fit index (IFI)	0–1	0.991
Normed fit index	Root mean squared error of approximation (RMSEA)	< 0.1	0.046
	Normed chi-square (CMIN/DF)	1–3	1.425
P value		> 0.05	0.071

PHSRA index was developed as follow:

8 $$ PHSRA=10\times \left[0.031\times \left(\frac{A-22.00}{33.00}\right)+0.145\times \left(\frac{BMI-19.32}{15.62}\right)-0.064\times \left(\frac{VO2\mathit{\max}-29.30}{14.86}\right)+0.106\times \left(\frac{BSA-1.64}{0.83}\right)+0.739\times \left(\frac{T_a-21.97}{26.23}\right)+0.688\times \left(\frac{T_w-12.10}{25.47}\right)+0.765\times \left(\frac{T_g-23.40}{39.30}\right)+0.245\times \left(\frac{V_a-0.0}{4.20}\right)+0.482\times \left(\frac{M_t-130}{360}\right)+0.383\times \left(\frac{I_c-0.50}{0.85}\right)\right] $$

where A is age (year), BMI is body mass index (kg/m^2^), VO2max is maximum aerobic capacity (ml/(kg·min)), BSA is body surface area (m^2^), T_a_ is dry temperature (^0^ C), T_w_ is wet temperature (^0^ C), T_g_ is globe temperature (^0^ C), V_a_ is wind speed (m/s), M_t_ is total metabolism (watts), and I_c_ is insulation of clothes (clo). In this equation, it is important to note that signs of coefficients of body surface area and wind speed change from positive (+ 0.106 and + 0.245) to negative (− 0.106 and − 0.245) when the dry temperature is below 35 ^0^ C (normal skin temperature) because the heat strain decreases in these conditions.

**Fig. 2:**
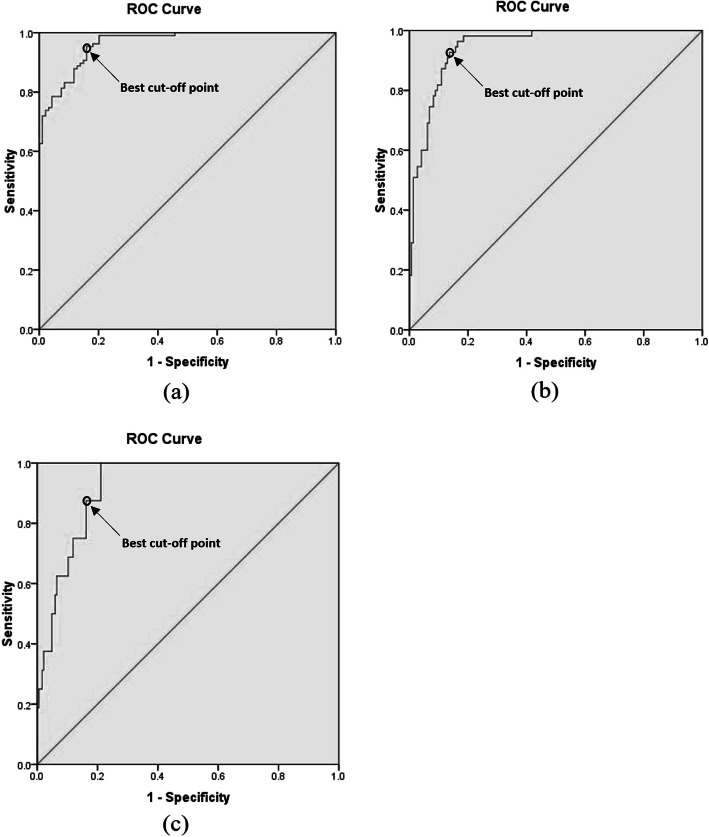
Receiver operating characteristic (ROC) curves of (**a**) low and moderate, (**b**) moderate and high, and (**c**) high and very high risk zones

**Fig. 3:**
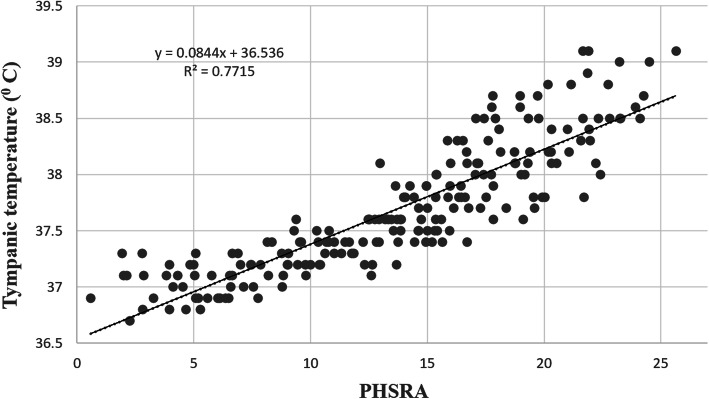
Linear regression curve between PHSRA index and core temperature

Figure 2 exhibits the Receiver operating characteristic (ROC) curves related to various risk zones. The empirical ROC curve is a plot of the true positive rate (sensitivity) versus the false positive rate (1 - specificity) for all possible cut-off values of PHSRA index, and best cut-off point has highest true positive rate and lowest false positive rate in diagnosing the intended risk level of thermal strain. In the present study, the PHSRA index was developed as an alternative to tympanic temperature in determining the risk of thermal strain. Therefore, these curves specify the optimal values of the PHSRA index equivalent to tympanic temperatures of 37.5, 38.0, and 38.5 °C, as boundaries of the different risk levels. The values of all possible cut-off points of the PHSRA index with their sensitivity and specificity are reported in the tables of coordinates of ROC curves. The values of optimal cut-off points were extracted from these tables. Based on the results, the optimal cut-off points between low and moderate risk, moderate and high risk, and high and very high risk were equal to 12.93 (sensitivity = 0.953 and specificity = 0.840), 16.48 (sensitivity = 0.927 and specificity = 0.863), and 18.87 (sensitivity = 0.875 and specificity = 0.838), respectively. The area under of ROC curves (AUC) was also calculated as 0.965 (95% CI: 0.944, 0.985) (*p* < 0.001), 0.947 (95% CI: 0.919, 0.976) (*p* < 0.001), and 0.923 (95% CI: 0.879, 0.967) (*p* < 0.001), respectively. Figure 3 also shows the linear regression curve between tympanic temperature and PHSRA index. As a result, this index justifies 77% of the tympanic temperature (R^2^ = 0.77).

## Discussion

The results showed that extensive ranges of values with normal distributions were collected. Therefore, the novel index extracted from these data is usable for the risk assessment of people with different body characterization in a variety of climatic conditions. In the model of the present study, the variables were divided into two groups, including main and personal factors. The results determined that the main factors with a total coefficient of 0.79 and personal factors with 0.14 had significant direct effects on the tympanic temperature. The results also indicated that the influence of the main factors in producing thermal strain compared to that of personal factors was significantly higher. The findings of other studies support these results. In a study performed by Golbabaei et al. [30], the impact coefficients of the task, environment, and worker variables on the thermal strain were calculated as 0.526, 0.279, and 0.195, respectively. In the study of Zheng et al. [31], the weights of the task, environment, and worker variables were obtained as 0.540, 0.297, and 0.163, respectively. These findings are consistent with those of the present study. However, as a result, personal factors make individual differences in susceptibility to heat disorders and illnesses and possess importance in thermal risk assessments. Alfano et al. [32] concluded that a two-dimensional environmental index such as humidex underestimated the body’s physiological response under warm conditions. The results of a study conducted by Potter et al. [33] showed that the use of a mathematical model developed by variables of the environment, activity, and clothing was restricted to general predictions of thermal strain and did not provide individual estimations obtained from physiological sensor data. Hence, the use of personal factors in the development of heat stress indices can make accurate predictions of the thermal strain.

The indirect effect coefficients of the personal factors including the body mass index, body surface, maximum aerobic capacity, and age were obtained as 0.145, 0.106, − 0.064, and 0.031, respectively. Brode et al. [34] also studied the effect of individual variables on the thermal comfort evaluated by the universal thermal climate index (UTCI). The results indicated that gender and age had a negligible influence on prediction error, whereas there was an increase in the error for obese people. In a review study performed by Kenny et al. [9], the odds ratio of heat-related death and or hospital admission at obese persons was calculated as 1.2. These results indicate the importance of obesity in assessing thermal risk. Additionally, body surface area plays an essential role in the regulation of tympanic temperature. However, its effect depends on the air temperature. Large body surface area increases the heat absorption in warm and hot environments while it decreases the thermal strain in cool environments. Of personal factors, VO2max had a reverse relationship with the tympanic temperature. These findings are in agreement with those of Havenith and Middendrop [35], who concluded a significant negative correlation between VO2max with heart rate and core temperature under various climatic conditions. Based on the results, the lowest effect coefficient was related to the variable of age. The results of other studies have been demonstrated that human physical characteristics such as aerobic capacity, body surface area, and fat percentage adjust the influence of age on the thermal strain. Wright et al. [36] concluded that different age groups had similar hydration, thermal, and cardiovascular response when they were matched in terms of demographic characteristics. It may be a reason for the obtained result in the current study. Moreover, the results showed that the environmental variables could impress on VO2max. Craig and Cummings [37] also observed that dehydration due to the activity under thermal conditions decreases the aerobic capacity (VO2max).

Of the main factors, the highest indirect effects were related to environmental variables, including globe temperature with a coefficient of 0.765, dry temperature with 0.739, and wet temperature with 0.688. The same results were obtained in the study of Dehghan et al. [38]. However, the coefficients of these variables in wet bulb globe temperature (WBGT) index are equal to 0.20, 0.10, 0.70, respectively [39]. These differences may be due to the use of normalized values in the PHSRA index and the use of non-normalized values in the WBGT index. Based on the results, the wind speed had the lowest impact coefficient among the main factors. This parameter was often unstable during measurements, which diminished its impact on the thermal strain.

Linear regression analysis was used to examine the validity of the novel index. The results showed that the PHSRA index could justify 77% of the tympanic temperature. In the study of Falahati et al. [40], this value for the WBGT index was equal to 57%. Malchaire [41] concluded that the PHS index predicted 66% of variations in the rectal temperature. The results of Monazzam et al. [42] study also showed that WBGT and PHS indices justified 71 and 76% of the aural temperature, respectively. These findings demonstrate that the PHSRA index has an appropriate validity for predicting the thermal strain. This validity may be due to the use of personal and main factors simultaneously in developing the index. As well as, the results showed that the presented model had good fitness, and the diagnostic accuracies of ROC curves were acceptable. A limitation of the present study was the general assessment of all environments by an index. Additionally, it was not possible to apply very accurate equipment such as rectal temperature because of ethical problems.

## Conclusion

In total, the present study showed that personal characteristics in addition to main agents, including environment, metabolism, and clothing, play an important role in producing thermal strain. Therefore, the developed index can be applied to screen and assess people with various personal properties for employing in a variety of thermal conditions. This index can predict the risk of thermal strain and prevent the occurrence of heat-related illnesses. The results indicated that the developed index had an acceptable validity in the prediction of thermal strain. However, it is suggested that this index is validated in various workplaces with thermal conditions. Its validity can also be investigated to use in non-work environments. Moreover, it is needed that a heat risk assessment index for women is separately developed in future researches.

## Data Availability

All data generated and analyzed during this step of study are included in this published article.

## Abbreviations

WBGT: Wet bulb global temperature
PHS: Predicted heat strain
UTCI: Universal thermal climate index
PHSRA: Personal heat strain risk assessment
BMI: Body mass index
VO2max: Maximum aerobic capacity
BSA: Body surface area
SEM: Structural equation modeling
ROC: Receiver operator curves
